# Transcriptome-wide analysis of RNA m^6^A methylation regulation of muscle development in Queshan Black pigs

**DOI:** 10.1186/s12864-023-09346-w

**Published:** 2023-05-04

**Authors:** Yaqing Dou, Yilin Wei, Zhe Zhang, Chenlei Li, Chenglei Song, Yingke Liu, Kunlong Qi, Xinjian Li, Xiuling Li, Ruimin Qiao, Kejun Wang, Feng Yang, Xuelei Han

**Affiliations:** College of Animal Science and Technology, Henan Agricultural University, Zhengzhou, 450046 China

**Keywords:** Queshan Black pig, Longissimus dorsi, MeRIP-Seq, Muscle growth and development, m^6^A-modified genes

## Abstract

**Background:**

N^6^-methyladenosine (m^6^A) refers to the methylation modification of N^6^ position of RNA adenine, a dynamic reversible RNA epigenetic modification that plays an important regulatory role in a variety of life processes. In this study, we used MeRIP-Seq and RNA-Seq of the longissimus dorsi (LD) muscle of adult (QA) and newborn (QN) Queshan Black pigs to screen key genes with m^6^A modification involved in muscle growth by bioinformatics analysis.

**Results:**

A total of 23,445 and 25,465 m^6^A peaks were found in the whole genomes of QA and QN, respectively. Among them, 613 methylation peaks were significantly different (DMPs) and 579 genes were defined as differentially methylated genes (DMGs). Compared with the QN group, there were 1,874 significantly differentially expressed genes (DEGs) in QA group, including 620 up-regulated and 1,254 down-regulated genes. In order to investigate the relationship between m^6^A and mRNA expression in the muscle of Queshan Black pigs at different periods, a combined analysis of MeRIP-Seq and RNA-Seq showed that 88 genes were significantly different at both levels. Gene Ontology and Kyoto Encyclopedia of Genes and Genomes results showed that DEGs and DMGs were mainly involved in skeletal muscle tissue development, FoxO signaling pathway, MAPK signaling pathway, insulin signaling pathway, PI3K–Akt signaling pathway, and Wnt signaling pathway. Four DEGs (*IGF1R*, *CCND2*, *MYOD1* and *FOS*) and four DMGs (*CCND2*, *PHKB*, *BIN1* and *FUT2*), which are closely related to skeletal muscle development, were selected as candidate genes for verification, and the results were consistent with the sequencing results, which indicated the reliability of the sequencing results.

**Conclusions:**

These results lay the foundation for understanding the specific regulatory mechanisms of growth in Queshan Black pigs, and provide theoretical references for further research on the role of m^6^A in muscle development and breed optimization selection.

**Supplementary Information:**

The online version contains supplementary material available at 10.1186/s12864-023-09346-w.

## Introduction

With the advancement of epigenetic studies, chemical RNA modifications have received increasing attention from researchers. To date, more than 150 types of RNA post-transcriptional modifications have been identified in all organisms [1]. The common modifications are N^6^-methyladenosine (m^6^A), N^1^-methyladenosine (m^1^A), 5-methylcytosine (m^5^C), and pseudouridine (ψ) [2]. In the 1970s, the presence of m^6^A modification was first identified in hepatocellular carcinoma cells by Desrosiers et al. [3]. m^6^A refers to the methylation modification of N^6^ position of RNA adenine, which occurs in the highly conserved consensus sequence RRACH (R = G or A; H = A, C, or U) [4] and is most enriched near the 3′ untranslated regions (UTRs), followed by the coding sequence (CDS) region and 5′ UTRs [5]. It is one of the most abundant and highly conserved forms of post-transcriptional modifications in eukaryotes and is widely present in mRNAs, tRNAs, rRNAs, mRNAs, and lncRNAs [6]; and plays a role in post-transcriptional regulation, affecting mRNA splicing, expression, translation, and stability [7, 8]. In addition, it is involved in a variety of biological processes, such as energy metabolism and adipogenesis [9].

To date, studies on m^6^A methylation modifications have focused on humans, mice, and other model animals; however, little is known about its specific mechanisms of action. Similar to DNA and histone methylation classes, m^6^A methylation is dynamically reversible in mammals [10] and relies on the co-regulation of multiple proteins, including m^6^A methyltransferases (writers), demethylases (erasers), and m^6^A methylated reading proteins (readers) [11, 12]. m^6^A methyltransferases, such as METTL3, METTL14, and WTAP are the core proteins of methyltransferases that form the m^6^A methyltransferase complex and play a catalytic role [13]. m^6^A-methylated methyl is derived from S-adenosylmethionine (SAM); METTL3 binds SAM and transfers the methyl to a specific site in the mRNA [14]. METTL3 exerts catalytic activity, whereas METTL14 does not have catalytic activity but can facilitate the binding of METTL3 to substrates. WTAP does not have methylation activity but can interact with the METTL3–METTL14 complex and participate in the m^6^A modification process together. Only two types of demethylases have been identified, namely, FTO and AlkB homolog 5 (ALKBH5). FTO is the earliest identified demethylase. ALKBH5 can remove methyl directly from m^6^A-modified adenine without going through the oxidation process and can effectively remove m^6^A modification from mRNA [15]. m^6^A-binding proteins mostly carry conserved YTH structural domains, such as the YTH homologous structural domain protein family (YTHDF1, YTHDF2, and YTHDF3) and YTH structural domain proteins (YTHDC1, YTHDC2). YTHDF1 is able to bind to m^6^A modification sites to improve mRNA translation efficiency, and YTHDF2 promotes mRNA degradation and dynamically regulates mRNA abundance [5]. YTHDF3 can further enhance mRNA translation or degradation by binding to YTHDF1 or YTHDF2 [16]. YTHDC1 is an intranuclear binding protein that is capable of participating in mRNA processing and nuclear localization. YTHDC2 possesses a specific helix and protein repeat structural domain where binding to the m^6^A modification site is achieved, which in turn promotes mRNA translation [17]. In addition to proteins containing the YTH structural domain, an increasing number of binding proteins play important functions in the m^6^A modification process.

Queshan Black pig is a local excellent pig breed with high fertility, adaptability, excellent meat quality, and stable genetic performance in Henan Province, China. Our laboratory has conducted long-term research around this breed to understand its specific physiological characteristics, growth, and development mechanism [18, 19]. Based on the indispensable role played m^6^A methylation modifications in regulating gene expression and participating in various biological processes, we hypothesized that m^6^A modifications are involved in the muscle growth and development of Queshan Black pigs. Understanding the molecular mechanisms of muscle growth is essential for maintaining meat yield and quality. Therefore, this study sequenced QN and QA LD samples by MeRIP-Seq and RNA-Seq to identify differential methylation peaks (DMPs) and differentially expressed genes (DEGs), so as to further explore their function and mechanism. This study may provide a theoretical basis for further research on the specific regulatory mechanisms of growth of Queshan Black pigs and the optimal selection of this breed.

## Materials and methods

### Ethics statement

All of the experiments involving animals were carried out in accordance with the guidelines for the care and use of experimental animals established by the Ministry of Science and Technology of the People’s Republic of China (Approval Number DWLL20211193). The animal study was reviewed and approved by the Ethics Committee of Henan Agricultural University. In addition, all experiments were conducted in accordance with the relevant approved guidelines and regulations during slaughter, sampling, and sample conservation.

### Animals and tissue collection

Three QA and three QN pigs, all male, were selected in this study. The Queshan Black pigs used in this experiment were all from Queshan Black pig breeding farm in Henan Province. Each group of pigs were fed under the same conditions. The piggery type is completely enclosed piggery, the breeding environment is suitable, the pigs are healthy, no genetic diseases. Piglets were slaughtered at 3 days of age with a weight of 2.2 − 2.5 kg. The body length of QN1 − QN3 were 34, 35 and 35 cm, respectively. Adult pigs were slaughtered at 270 days of age and weighing 100 − 102 kg. The body length of QA1 − QA3 were 103, 111 and 103 cm, respectively. The longissimus dorsal (LD) muscle between the 6th and 7th ribs was collected and immediately stored in liquid nitrogen. Afterward, the muscle samples were placed at − 80 °C for subsequent experiments.

### RNA isolation, library preparation and sequencing

Total RNA was isolated and purified using TRIzol reagent (Invitrogen, Carlsbad, CA, USA) following the manufacturer's procedure. The total RNA quality and quantity were determined by Bioanalyzer 2100 and RNA 6000 Nano LabChip Kit (Agilent, CA, USA) with RIN number > 7.0. Poly(A) RNA was specifically captured from 50 μg total RNA using Oligo-dT magnetic beads and fragmented using Magnesium RNA Fragmentation Module (NEB, cat.e6150, USA). The cleaved RNA fragments were incubated for 2 h at 4 °C with m^6^A-specific antibody (No. 202003, Synaptic Systems, Germany) in IP buffer (50 mM Tris–HCl, 750 mM NaCl, and 0.5% Igepal CA-630) supplemented with BSA (0.5 mg/ ml). The eluted RNA was precipitated with 75% ethanol. According to the chain-specific library prepared by the dUTP method, the eluted m^6^A fragment (IP) and the unprocessed input control fragment were converted into the final cDNA library. The average insert size of the paired-end libraries was 100 ± 50 bp. Lastly, we performed paired-end sequencing (PE150) on an Illumina Novaseq™ 6000 platform (LC-Bio Technology Co., Ltd., Hangzhou, China) following the vendor's recommended protocol.

### Bioinformatics analysis process

Fastp software (v0.19.4, https://github.com/OpenGene/fastp) was used to remove the reads with adaptor contamination, low-quality bases, and undetermined bases with default parameter [20]. The sequence quality of the IP and input samples were verified using FastQC (https://www.bioinformatics.babraham.ac.uk/projects/fastqc/) and RseQC (http://rseqc.sourceforge.net/) [21, 22]. Then, HISAT2 (v2.0.4, http://daehwankimlab.github.io/hisat2) was used to map reads to the *Sus scrofa* (version 11.1) [23]. Peak calling and diff peak analysis were performed by R package exomePeak2 (v1.5.0, https://bioconductor.org/packages/release/bioc/html/exomePeak2.html). The threshold settings of differential peak and differential expression were |log2FC|≥ 1 and *p*-adjust < 0.05. and peaks were annotated by intersection with gene architecture using R package ANNOVAR (http://www.openbioinformatics.org/annovar/) [24, 25]. MEME (v5.3.3, http://meme-suite.org) and HOMER (v4.10, http://homer.ucsd.edu/homer/motif) were used for de novo and known motif finding, followed by motif localization with respect to the peak summit [26, 27]. StringTie (v2.1.2, https://ccb.jhu.edu/software/stringtie) was used to determine the expression levels of all transcripts and genes from input libraries by calculating the FPKM (total exon fragments / mapped reads [millions] × exon length [kB]) [28]. The differentially expressed transcripts and DEGs were selected with |log2FC|≥ 1 and *p*-adjust < 0.05 by R package edgeR (v4.1, https://bioconductor.org/packages/edgeR) [29]. The gene ontology (GO) and Kyoto Encyclopedia of Genes and Genomes (KEGG) pathway enrichments of DEGs were performed by KOBAS (v3.0) online analyses. *P* < 0.05 was considered statistically significant. All KEGG pathways shown in the manuscript can be found on the official KEGG website [30-32]. Protein − protein interaction (PPI) analysis was performed for DEGs and DMGs. The selected genes were imported into STRING (v11.5) for online analysis, and corresponding data were imported into Cytoscape (v.3.9.1) for visualization. Cytoscape (v.3.9.1) was also used to visualize the network of pathways and genes. Gene set enrichment analysis (GSEA) was performed using the OmicStudio tools at https://www.omicstudio.cn/tool. The pathway with |NES|> 1, NOM *p*-val < 0.05, FDR *q*-val < 0.25 was considered statistically significant.

### MeRIP-qPCR

According to the manufacturer's instructions, total RNA was extracted from the LD muscle tissue, the RNA fragments were converted into ~ 200nt fragments using the riboMeRIP™ m^6^A Transcriptome Profiling Kit (10 Assay) (RN: R11096.6, RiboBio, Guangzhou, China), and anti-m6A magnetic beads were prepared. Part of the RNA samples were taken as Input, and the rest were used as IP group for immunoprecipitation. Protein A/G magnetic beads were added into IP group and incubated at 4℃. Then the MagenTM Hipure Serum/plasma miRNA kit (R4317-03, Magen, Guangzhou,China) was used for elution and RNA recovery, and the obtained RNA was used for subsequent reverse transcription and q-PCR verification.

### Quantitative Real-Time PCR

We verified four DEGs and four DMGs to study the status of m^6^A in LD tissues of Queshan Black pigs at different periods. RNA extracted from muscle was reverse-transcribed into cDNA using the Evo M-MLV RT Kit with gDNA Clean for qPCR kit (AG11705, Accurate Biotechnology (Hunan) Co.,Ltd, Changsha, China). q-PCR was performed using the SYBR Green Premix Pro Taq HS qPCR Kit (AG11701, Accurate Biotechnology (Hunan) Co.,Ltd, Changsha, China) using the CFX96 real-time PCR detection system (Thermo Fisher Scientific, USA) according to the instructions. The *GAPDH* was used as the internal reference gene to normalize the gene expression levels. The relative expression of genes was calculated using the 2^−ΔΔCt^ method. MeRIP-qPCR does not require internal reference genes, and the IP/input ratio is calculated by 2^−ΔCt^ (ΔCt = Ct_IP_ − Ct_input_) and the ratio of IP RNA template and input RNA template to initial RNA when reverse transcription is introduced. Detailed primers are shown in supplementary Table 1 (Table S1).

### Statistical analysis

The data are expressed as mean ± standard deviation (*n* = 3). The student’s t-test and one-way analysis of variance (ANOVA) was performed using GraphPad Prism 8 software to determine the significance of differences between comparison groups. The difference between the means was considered statistically significant when *p*-value ≤ 0.05.

## Result

### Sequence statistics, quality control and reference genome alignment

After using fastp to filter out unqualified sequences from the raw data, the clean data was used for MeRIP-Seq and RNA-Seq analysis. We obtained two sets of QA and QN muscle sample data readings with three biological replicates per set. For the valid data of each group of samples, base proportion with mass value ≥ 20 (sequencing error rate less than 0.01), base proportion with mass value ≥ 30 (sequencing error rate less than 0.001) and GC content proportion are shown in Table S2.

The IP samples from the longissimus dorsi (LD) muscles of QA and QN pigs are denoted as QA_IP and QN_IP, respectively, in the m^6^A-Seq library in Figure S1. The LD muscle samples from QA and QN pigs are denoted as QA_input and QN_input in the RNA-Seq library, respectively. Each set of samples was repeated three times. The unique mapped reads are shown in Table S3. According to the regional distribution information of the reference genome, which can be defined compared with exon, intron, and spacer regions, the percentage contents of sequenced sequences localized to exon regions should be the highest under normal conditions. The analysis results are shown in Figure S1.

### Identification of m^6^A modification sites and analysis of differential methylation peaks

**Fig. 1:**
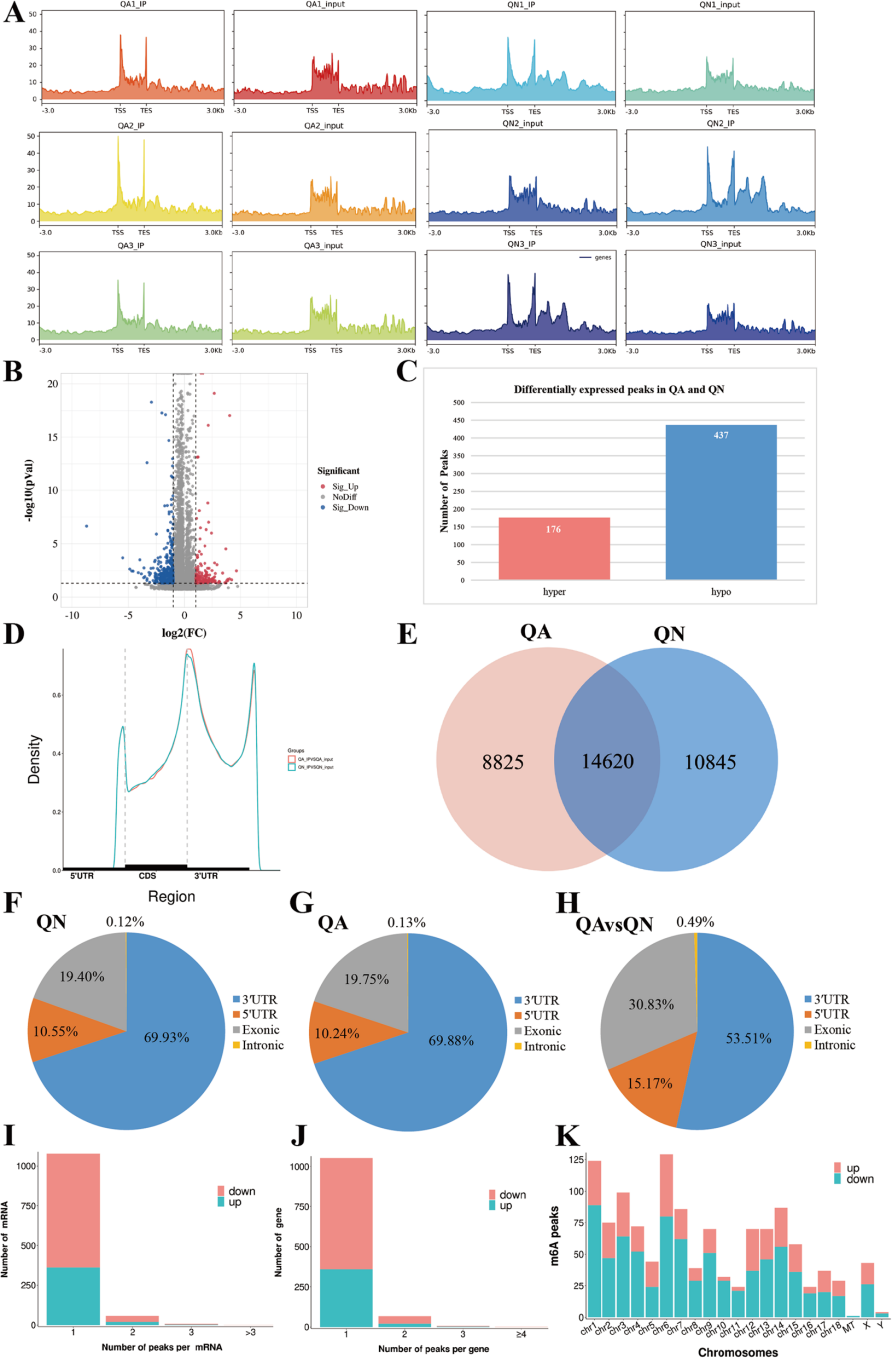
Overview of altered m6A-modified transcripts in Queshan Black pig LD muscle. **A** Enrichment of reads near TSS at the transcriptome initiation site of the genes. **B** Volcano plots showing significantly different m6A peaks. **C** Number of up- and downregulated DMPs. **D** Metagene plots displaying the regions of m6A peaks identified across the transcripts in QA and QN groups. **E** The number of common and specific m6A peaks in QA and QN groups. **F**–**G** Distribution of m6A peaks in QA and QN groups. **H** Distribution of DMPs. **I** Distribution of altered m6A peaks per mRNA. **J** Distribution of altered m6A peaks per gene. **K** Distribution of altered m6A peaks in human chromosomes

Based on MeRIP-seq (IP) and RNA-seq (Input) sequencing data, the position information and length information of peaks on the genome were obtained. Reads near TSS were abundant at the transcriptome start of genes, and the peak distribution is shown as a heat map in Fig. 1A. Next, with *p*-adjust < 0.05 and |log2FC|≥ 1 for threshold selection differential methylation peaks (DMPs) and differentially methylated genes (DMGs). A total of 613 DMPs were screened and 579 DMGs were annotated in the QA group compared with the QN group (Table S4). A total of 176 peaks showed increased expression (corresponding to 167 genes upregulated in m^6^A abundance), and 437 peaks showed decreased expression (corresponding to 418 genes downregulated in m^6^A abundance) as shown in Figs. 1B and C. The m^6^A peaks in QA and QN were enriched in the CDS near the stop codon (Fig. 1D). Through exomePeak2 analysis, 8,825 and 10,845 peaks were specifically expressed in the QA and QN groups, respectively, and 14,620 peaks were common between the two groups (Fig. 1E). The transcription products were divided into four regions, namely, 5′ UTR, 3′ UTR, exonic region, and intronic region. The distribution of m^6^A peaks in QA and QN was similar (Figs. 1F and G). The analysis of DMPs enrichment sites showed that 53.51% of the DMPs were enriched in the 3′ UTR, about 30.83% were in the exonic region, and 15.17% of the m^6^A modifications occurred in the 5′ UTR (Fig. 1H). By analyzing the distribution of m^6^A peaks for each mRNA or gene, we found that most of the mRNAs or genes had one m^6^A peak (mRNAs with upregulated peaks: 362/384, mRNAs with downregulated peaks: 714/758, genes with upregulated peaks: 360/383,; genes with downregulated peaks: 693/747. Figs. 1I and J). All variable m^6^A peaks were mapped to human chromosomes. The presence of m^6^A peaks was found in all chromosomes, especially chr1, chr3, and chr6 (Fig. 1K). In Table 1, the differential m^6^A peaks were concentrated in the 3′ UTR. The Table 1 showed the top 20 differential m^6^A peaks, where log2FC < 0 represents hypomethylation, and log2FC ≥ 0 represents hypermethylation.

**Table 1:** The top 20 differentially expressed m^6^A peaks

Gene name	Log2FC	Regulation	Chromosome	Peak region	Peak star	Peak end	*p*-adj
USP49	4.66	Hypermethylation	7	3′ UTR	36,984,082	36,984,282	0.00
ENSSSCG00000041922	4.23	Hypermethylation	17	5′ UTR	48,666,269	48,666,369	0.02
MYO19	4.05	Hypermethylation	12	3′ UTR	38,024,950	38,029,720	0.00
CFAP70	4.04	Hypermethylation	14	3′ UTR	76,075,293	76,075,468	0.02
PRSS36	3.89	Hypermethylation	3	5′ UTR	17,345,869	17,346,919	0.03
FAM217B	3.87	Hypermethylation	17	5′ UTR	59,967,918	59,969,185	0.02
ENSSSCG00000028892	3.78	Hypermethylation	6	3′ UTR	92,429,402	92,429,680	0.03
SATB2	3.71	Hypermethylation	15	3′ UTR	102,964,778	102,965,078	0.05
CLK1	3.71	Hypermethylation	15	5′ UTR	104,561,110	104,561,260	0.00
RIMKLB	3.62	Hypermethylation	5	3′ UTR	62,683,415	62,683,540	0.05
ENSSSCG00000039926	− 8.71	Hypomethylation	Y	3′ UTR	6,511,355	6,511,580	0.00
ENSSSCG00000033122	− 5.50	Hypomethylation	6	CDS	61,825,045	61,825,620	0.00
ENSSSCG00000041158	− 4.87	Hypomethylation	1	5′ UTR	99,841,915	99,842,090	0.00
SLC9A1	− 4.69	Hypomethylation	6	CDS	84,385,735	84,385,835	0.00
PIDD1	− 4.57	Hypomethylation	2	CDS	502,063	502,452	0.00
ENSSSCG00000041218	− 4.11	Hypomethylation	13	CDS	9,844,590	9,872,000	0.01
ENSSSCG00000038327	− 3.97	Hypomethylation	6	CDS	61,825,266	61,825,816	0.00
PLEKHA5	− 3.84	Hypomethylation	5	CDS	53,781,210	53,782,987	0.01
RIMKLB	− 3.63	Hypomethylation	5	3′ UTR	62,679,815	62,679,915	0.02
ENSSSCG00000051287	-3.61	Hypomethylation	3	CDS	17,778,552	17,778,702	0.00

### Motif analysis

**Fig. 2:**
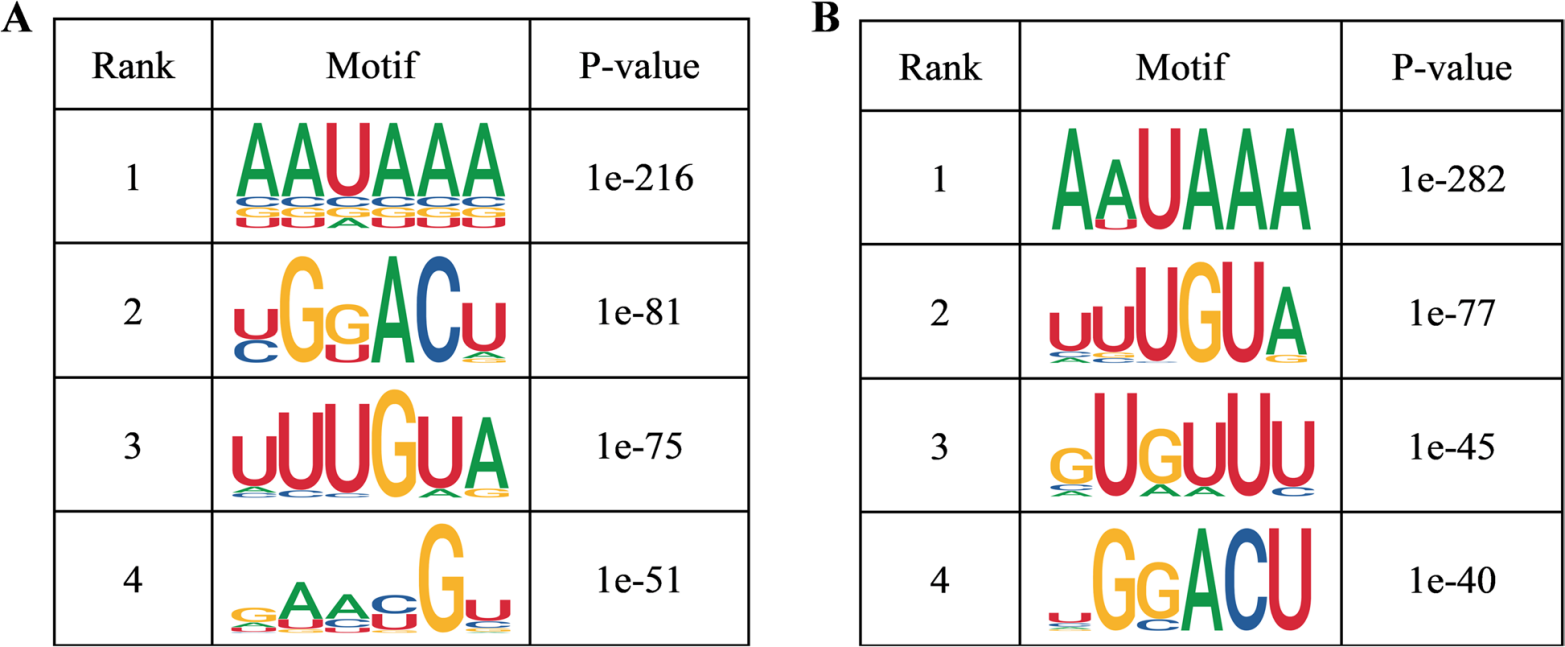
Sequence showing the motifs with significant differences in muscle samples at the m6A peak. **A** Top four motifs in the QA group. **B** Top four motifs in the QN group

RNA methylation and demethylation are initiated by the combined action of multiple binding proteins to the motifs of methylation sites. A motif is a biologically important nucleic acid sequence pattern that is highly conserved. We performed motif prediction for the two groups of samples and ranked them according to *p*-value. The smaller the *p*-value, the higher the ranking (Fig. 2). A common motif structure in RNA modification is RRACH (where R = A or G; H = A, C, or U).

### Enrichment analysis of differentially methylated genes

**Fig. 3:**
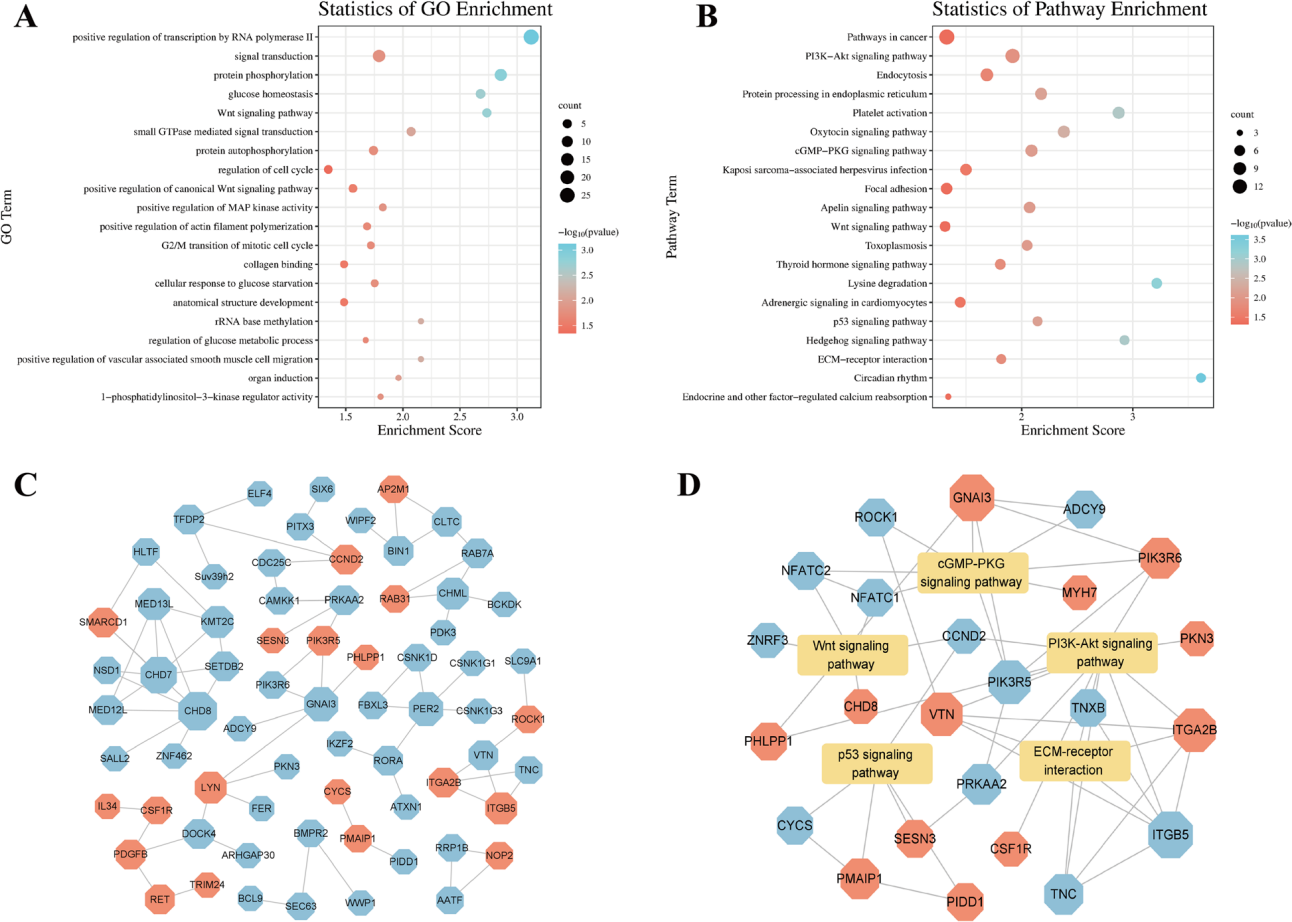
DMGs functional enrichment analysis. **A** GO enrichment terms and **B** KEGG analysis of DMGs. **C** PPI analysis of DMGs. **D** Pathways and DMGs network diagram. Octagonal nodes represent DMGs, rectangular nodes represent pathways, red represents up-regulation, blue represents down-regulation

GO and KEGG pathways of DMGs were performed to analyse the potential function of m^6^A-modified genes in the skeletal muscle growth and development of Queshan Black pigs. The enriched GO terms for the DMGs mainly included protein phosphorylation, regulation of glucose metabolic process, positive regulation of actin filament polymerization and regulation of cell cycle (Fig. 3A, Table S5). KEGG pathway enrichment analysis showed that DMGs were enriched to the PI3K–Akt signaling pathway, Wnt signaling pathway, p53 signaling pathway, Thyroid hormone signaling pathway, ECM-receptor interaction and other pathways related to myogenesis and development (Fig. 3B, Table S6). PPI analysis was performed for DMGs in GO terms and KEGG pathways shown in Figs. 3A and B. As shown in Fig. 3C, larger nodes indicate more connections. The network diagram of partial pathways and DMGs is shown in Fig. 3D. The results indicate that DMGs have a potential role in regulating gene expression and biological metabolism during the skeletal muscle growth and development of Queshan Black pigs.

### Analysis of differentially expressed genes

**Fig. 4:**
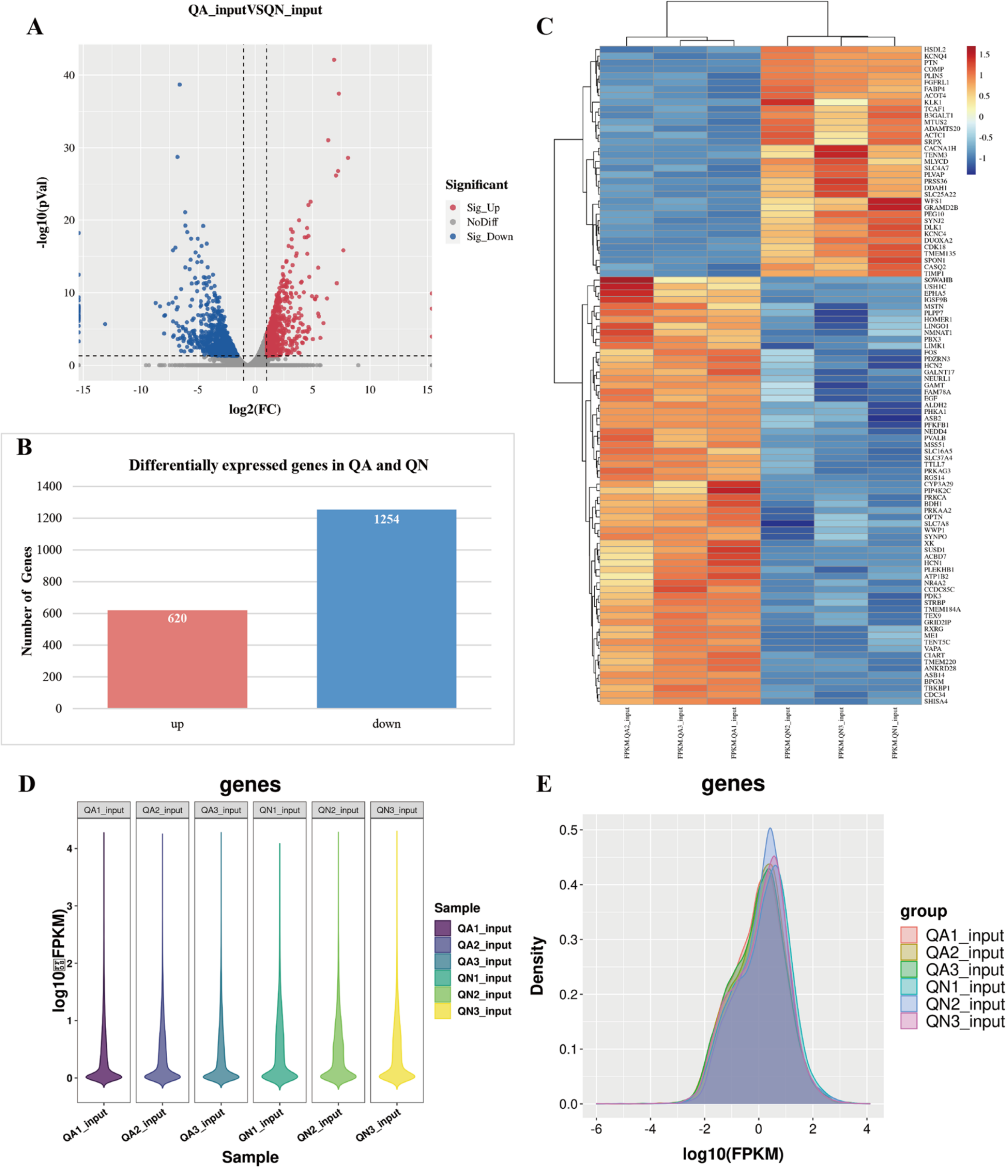
DEG analysis between QA and QN. **A** DEGs expression volcano diagram. **B** Number of up- and downregulated DEGs. **C** Heat map of DEGs. **D** Violin diagram of gene expression. **E** Density diagram of gene expression

RNA-Seq analysis was performed in all Input samples, and the overall distribution of DEGs could be understood through the volcano map in Fig. 4A. In this study, FPKM was used to measure the abundance of gene expression in different samples. As shown in Fig. 4B, this study found that among the 19,023 genes identified in the two groups of samples, 1,874 genes were differentially expressed in the QA group compared with the QN group, including 620 upregulated genes, 1254 downregulated genes (|log2FC|≥ 1 and *p*-adjust < 0.05, Table S7), and 17,149 genes without remarkable difference. As shown in Fig. 4C, the clustering patterns of genes between samples in two periods. Gene expression and expression density plots are shown in Figs. 4D and E, respectively. The top 20 DEGs are shown in Table 2.

**Table 2:** The top 20 differentially expressed genes

Gene name	Fold change	Regulation	Locus	Strand	*P*-adj
ACBD7	270.63	up	chr10, 46,740,585–46,750,606	+	0.00
ETNPPL	205.15	up	Chr8, 113,363,049–113,384,783	+	0.00
PVALB	157.04	up	chr5, 10,955,491–10,973,289	+	0.00
ENSSSCG00000044722	148.78	up	chr13, 75,843,255–75,845,051	+	0.00
PIP4K2C	138.18	up	chr5, 22,854,137–22,867,037	+	0.00
ENSSSCG00000033029	131.34	up	chr4, 86,919,184–86,929,920	+	0.00
ASB14	117.16	up	chr13, 39,123,650–39,146,914	−	0.00
ENSSSCG00000045624	81.99	up	chr1, 30,776,427–30,802,130	+	0.00
ENSSSCG00000047072	76.60	up	chr8, 3,764,912–3,769,144	−	0.00
SERPINB11	61.84	up	chr1, 158,015,718–158,030,763	−	0.00
IL18BP	0.00	Down	chr9, 6,488,808–6,591,333	+	0.00
PCK2	0.00	Down	chr7, 75,187,194–75,197,733	−	0.00
RXFP2	0.00	Down	chr11, 8,266,716–8,321,022	+	0.00
TRPV3	0.00	Down	chr12. 49,635,927–49,671,613	−	0.00
STK33	0.00	Down	chr9, 786,509–936,723	+	0.00
SLC6A13	0.00	Down	chr5, 67,577,197–67,612,681	−	0.00
CPLX1	0.01	Down	chr8, 197,881–232,038	−	0.00
ADAMTS19	0.01	Down	chr2, 132,145,389–132,409,915	+	0.00
ACTC1	0.01	Down	chr1, 136,281,167–136,286,545	+	0.00
SLF2	0.01	Down	chr14, 111,996,760–112,068,251	+	0.00

**Fig. 5:**
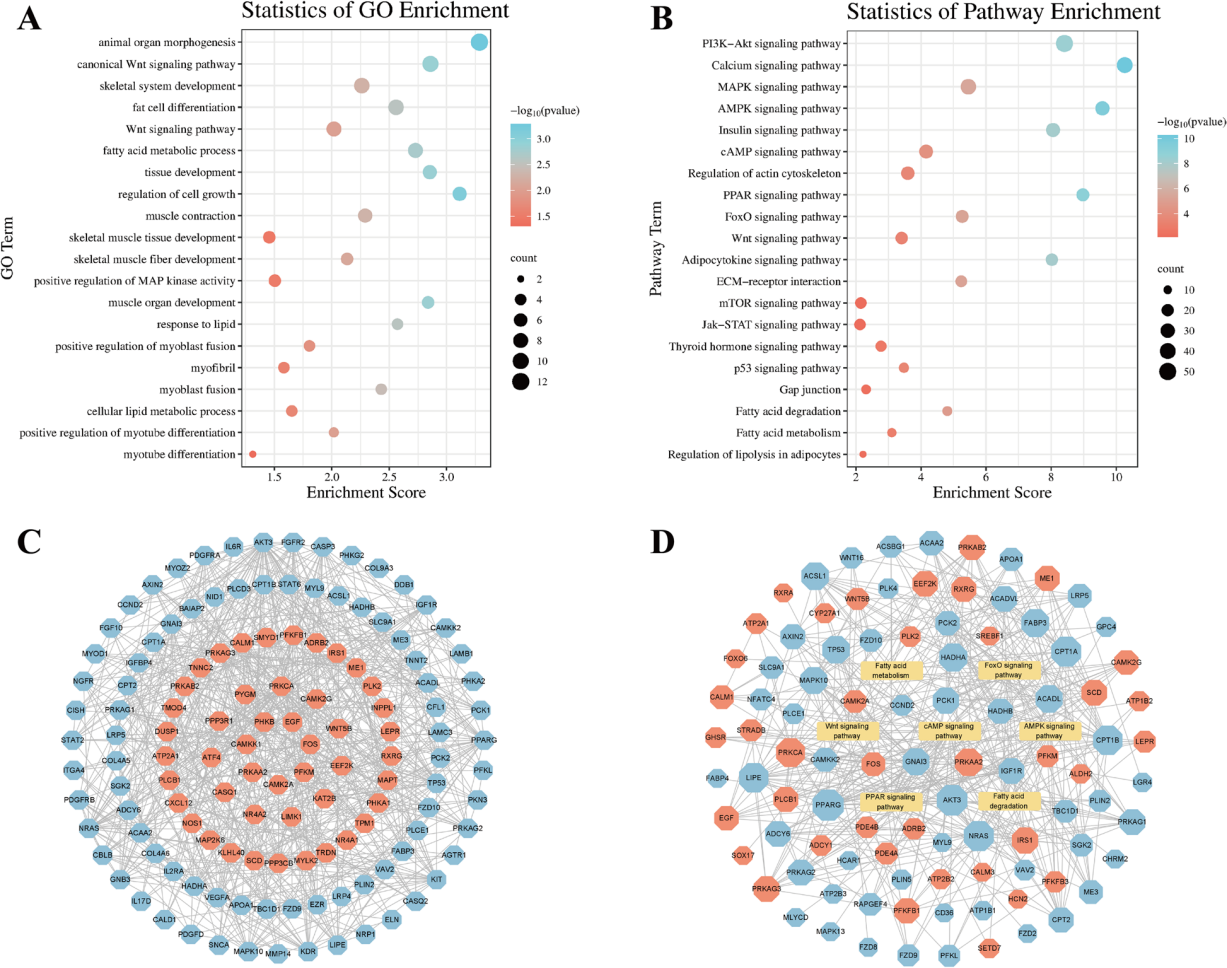
DEGs functional enrichment analysis. **A** GO enrichment terms and **B** KEGG analysis of DEGs. **C** PPI analysis of DEGs. **D** Pathways and DEGs network diagram. Octagonal nodes represent DEGs, rectangular nodes represent pathways, red represents up-regulation, blue represents down-regulation

GO and KEGG analyses were performed to further reveal the functions of DEGs. GO enrichment analysis of DEGs showed that including muscle organ development, fatty acid metabolic process, fat cell differentiation, muscle contraction, skeletal muscle tissue development and myotube differentiation, were significantly enriched (Fig. 5A, Table S8). KEGG analysis showed that DEGs were significantly enriched in such as PPAR signaling pathway, calcium signaling pathway, AMPK signaling pathway, Fatty acid degradation, Fatty acid metabolism, FoxO signaling pathway, MAPK signaling pathway, mTOR signaling pathway, Wnt signaling pathway, and other pathways related to fat deposition and muscle development regulation (Fig. 5B, Table S9). As shown in Fig. 5C, PPI analysis was performed for DEGs in GO terms and KEGG pathways shown in Figs. 5A and B. The network diagram of partial pathways and DEGs is shown in Fig. 5D.

### A conjoint analysis of MeRIP-Seq and RNA-Seq data

**Fig. 6:**
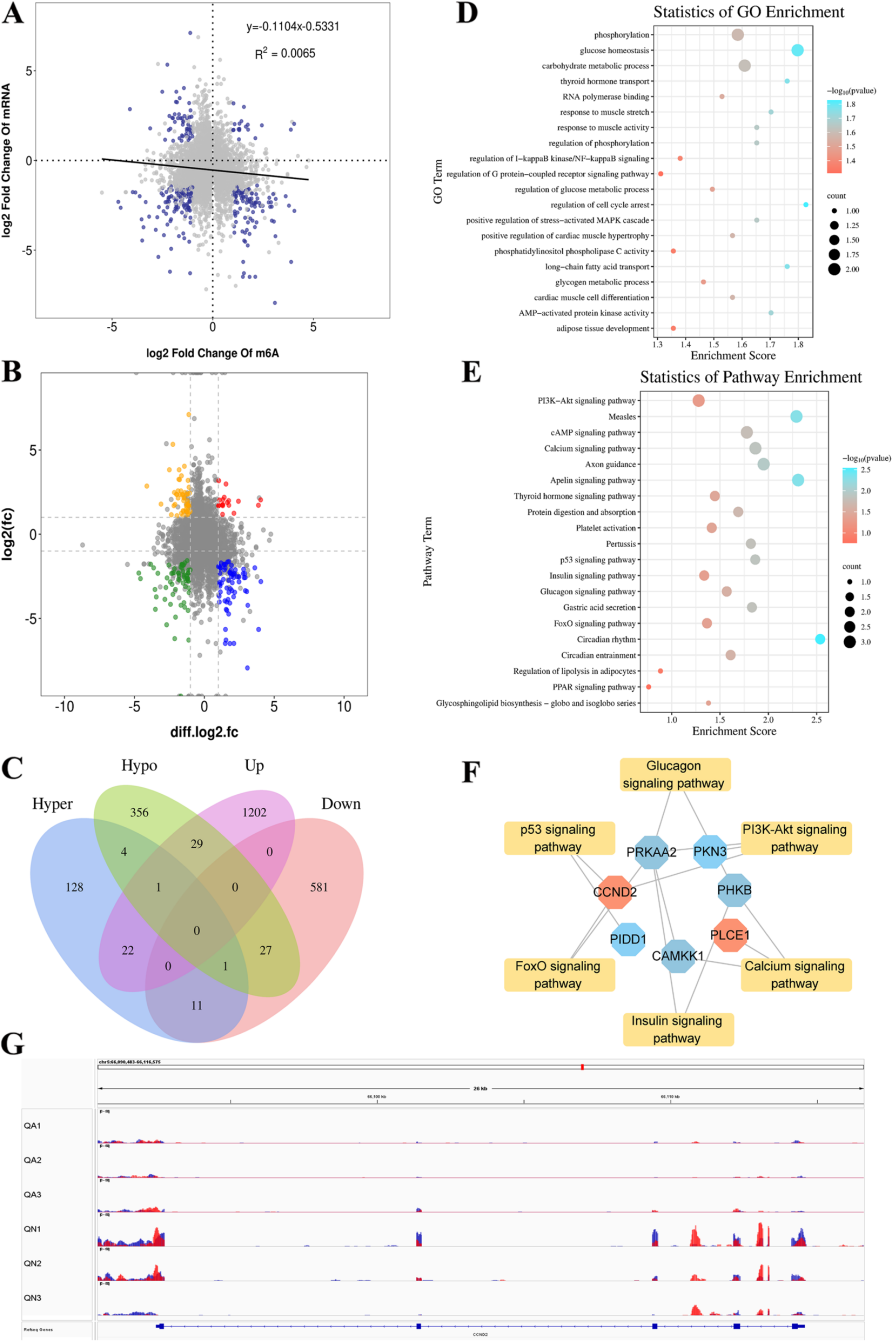
Results of MeRIP-Seq and RNA-Seq. **A** Correlation analysis diagram between methylation level and gene expression level. **B** Four-quadrant diagram of DMGs and DEGs. **C** Venn diagram of DMGs and DEGs. **D** GO enrichment terms and **E** KEGG analysis of codifferential genes (intersection genes of DMGs and DEGs). **F** Pathways and codifferential genes network diagram. **G** m6A enrichment and gene expression profile of CCND2 in QA and QN. Octagonal nodes represent codifferential genes, rectangular nodes represent pathways, red represents hyper-down, blue represents hypo-up, light blue represents hypo-down

In order to explore the potential regulatory effect of m^6^A modification on gene expression during skeletal muscle growth and development, the data of m^6^A-Seq and RNA-Seq were jointly analyzed in this study to further screen genes with substantial changes at the mRNA and m^6^A levels. The results revealed a negative correlation between methylation peak and gene expression level (Fig. 6A). This study found that 11,491 genes were modified by m^6^A in the QA group and 12,384 genes were modified by m^6^A in the QN group, among which 1,874 genes were significantly differentially expressed. Based on the results, 88 genes were screened out with remarkable alterations at both levels (Fig. 6B, Table S10). This result implies that m^6^A modifications may affect the expression of these genes during muscle growth and development. The overlapping results of DEGs and DMGs are shown in Fig. 6C, including 22 genes in common between "m^6^A_up" and "mRNA_up" (hyper-up) and 11 genes in common between "m^6^A_up" and "mRNA_down" (hyper-down). 29 genes (hypo-up) were common in "m^6^A_down" and "mRNA_up" and 27 genes (hypo-down) were common in "m^6^A_down" and "mRNA_down".

GO analysis showed that these genes were enriched in terms, such as long-chain fatty acid transport, thyroid hormone transport, response to muscle activity, adipose tissue development and regulation of I-kappaB kinase/NF-kappaB signaling (Fig. 6D, Table S11). KEGG pathway enrichment analysis showed that these genes were enriched in Calcium signaling pathway, FoxO signaling pathway, insulin signaling pathway, and PI3K–Akt signaling pathway and Thyroid hormone signaling pathway, which are remarkably associated with muscle development (Fig. 6E, Table S12). The network diagram of partial pathways and codifferential genes is shown in Fig. 6F. The differentially methylated sites in QA and QN showed altered intensity around the corresponding m^6^A peaks, according to Integrative Genomics Viewer (IGV) software (Fig. 6G).

### Gene set enrichment analysis

**Fig. 7:**
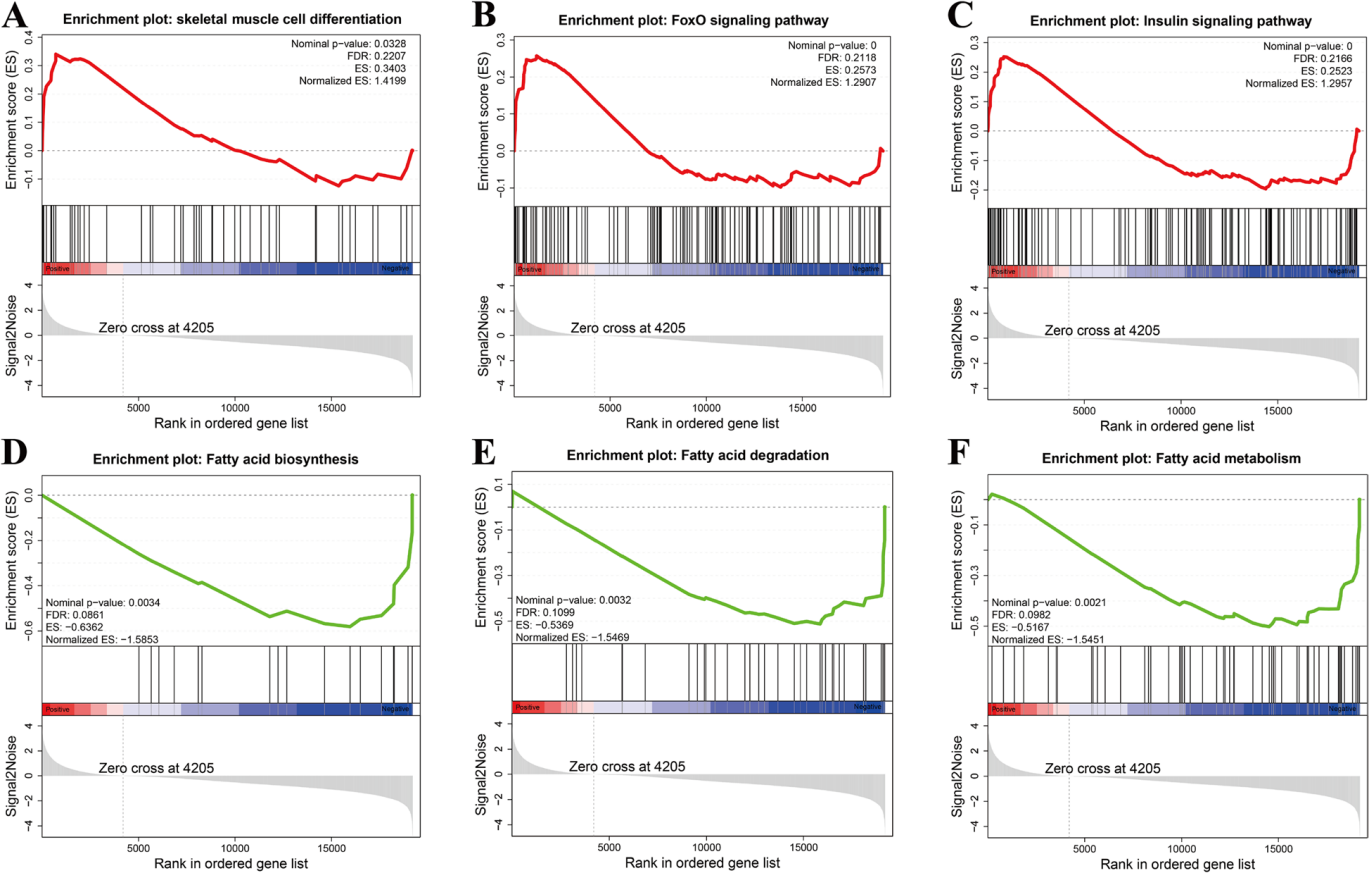
Gene set enrichment analysis (GSEA) indicated significant enrichment in **A** skeletal muscle cell differentiation. **B** FoxO signaling pathway. **C** Insulin signaling pathway. **D** Fatty acid biosynthesis. **E** Fatty acid degradation and **F** Fatty acid metabolism

The results of gene set enrichment analysis (GSEA) showed that it was consistent with the above KEGG enrichment results, skeletal muscle cell differentiation, Insulin signaling pathway and FoxO signaling pathway were also highly enriched (Fig. 7A-C). In addition, Fatty acid-related Fatty acid biosynthesis, Fatty acid degradation and fatty acid metabolism were also significantly enriched (Fig. 7D-F).

### Validation by MeRIP-qPCR and qRT-PCR

**Fig. 8:**
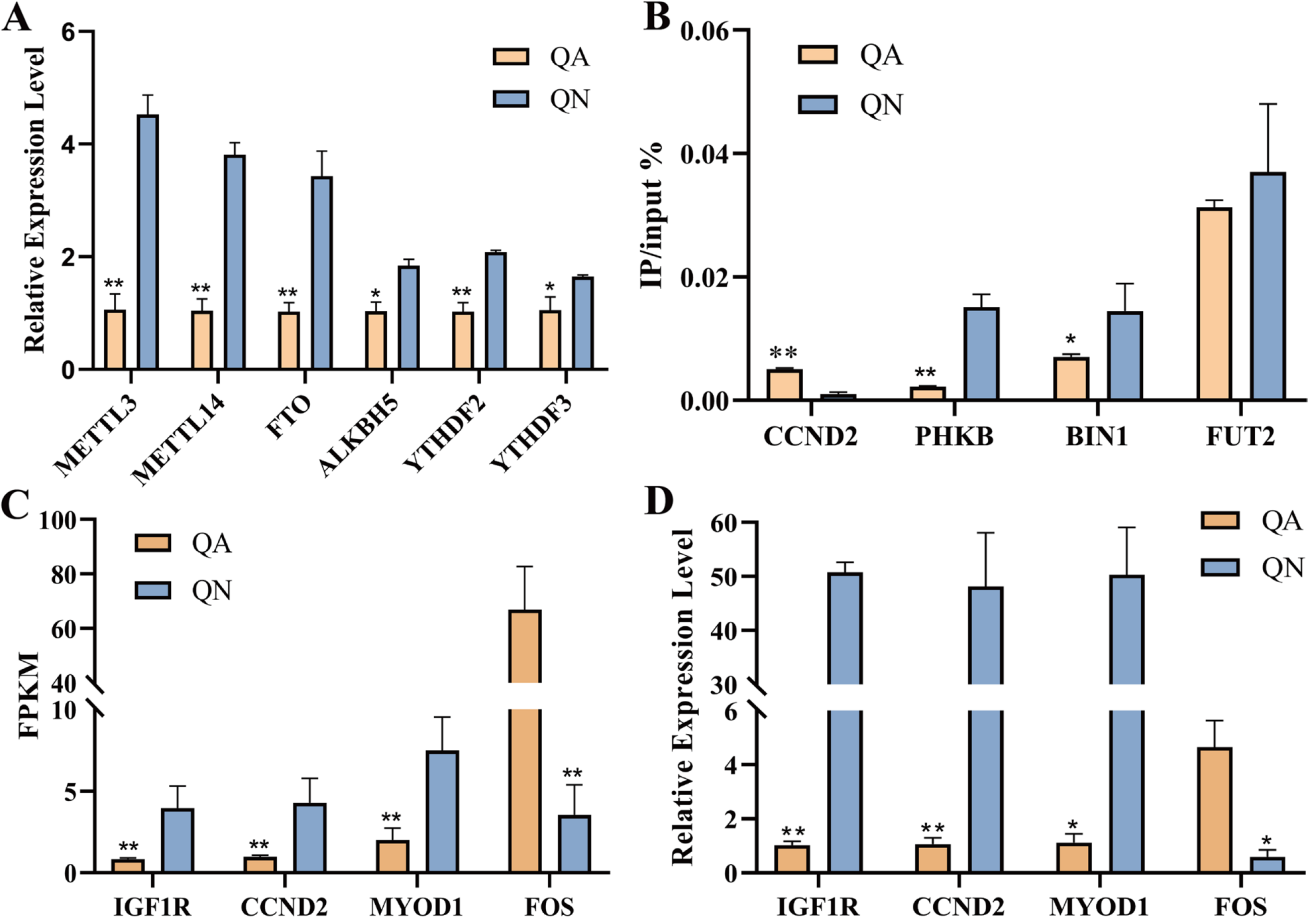
Experimental result. **A** Expression levels of methylation-related enzymes in QA and QN. **B** Methylase gene level was detected by MeRIP-qPCR. **C** Gene change levels based on RNA-Seq data. **D** The mRNA relative expression level was detected by qRT-PCR. ** represents *p* < 0.01, * represents *p* < 0.05

As shown in Fig. 8A, qRT-PCR was used in this study to detect the expression levels of methylation-related enzymes in QA and QN. The expressions of methylated transferase *METTL3*, *METTL14*, demethylated enzyme *FTO*, *ALKBH5* and methylated reading protein *YTHDF2*, *YTHDF3* in QN were significantly higher than those in QA group. In order to verify the accuracy of the sequencing results, four DMGs and four DEGs that may have potential regulatory relationships with muscle growth and development were screened in this study (Table 3), among which *CCND2* showed significant differences at both levels. The methylation level of DMGs and the gene expression level of DEGs were detected by MeRIP-qPCR and qRT-PCR respectively (Fig. 8B-D). The expression trends obtained by the experiment were consistent with the sequencing results. The consistency of the results confirmed the existence of m^6^A modification in the muscle of Queshan Black pig, which verified the reliability of the sequencing data.

**Table 3:** Sequencing results of candidate genes

Gene name	Pattern	Fold change	
		**m**^**6**^**A level**	**mRNA level**
IGF1R	Hyper-down	1.66	0.21
CCND2	Hyper-down	4.65	0.23
MYOD1	Hyper-down	1.49	0.27
FOS	Hypo-up	0.45	18.88
PHKB	Hypo-up	0.45	3.29
BIN1	Hypo-up	0.33	2.54
FUT2	Hypo-up	0.41	5.12

## Discussion

With the rapid development of science and technology in recent years, RNA methylation research has become a cutting-edge research hotspot in the field of epigenetics based on DNA and protein modification research. RNA m^6^A methylation is a highly conserved epigenomic modification that is widely found in various eukaryotes, such as yeast, plants, *drosophila*, and mammals, but has been studied rarely in livestock production animals, such as pigs. RNA m^6^A modifications can play a variety of biological functions; it is involved in disease development, stem cell differentiation, mRNA metabolism, animal energy metabolism, fat deposition, muscle growth and development [33-37]. In 2017, Tao et al. constructed the first transcriptome-wide m^6^A methylation modification map of porcine muscle tissue with the help of methylation RNA IP and MeRIP-Seq techniques, revealing that m^6^A methylation modification sites are distributed in the CDS region, stop codon, and 3′ UTR region in the porcine transcriptome [35]. It has been previously reported that m^6^A peaks are enriched near the stop codon in human transcripts, and many methylation sites are conserved in mouse and human transcripts [37]. The methylation modification sites in yak are concentrated in the stop codon, CDS, TSS, 3′ UTR, and, to some extent, in the 5′ UTR [38]. The results of the present study are consistent with this finding, as methylation modifications were located near the stop codon and in the 3′ UTR. Thus, these results suggest that the overall distribution of m^6^A modification sites is similar in the mammalian transcriptome, which again demonstrates that m^6^A modifications are conserved among species. In addition, our data showed that a large number of m^6^A methylation modifications existed in muscle tissue during the growth of the Queshan Black pig, which may have important effects on muscle fiber type, muscle cell maturation, muscle structural changes and further play an important regulatory role in the muscle growth and development of pigs by regulating gene expression levels.

RNA m^6^A modifications may play a key regulatory role in the differentiation and development of animal cells; for example, genes consistently modified by m^6^A are associated with myoblast growth and differentiation during three different developmental stages in yak [35]. In the transcriptome of embryonic stem cells, m^6^A-modified genes are involved in the regulation of embryonic stem cell pluripotency [7]. Therefore, in Queshan Black pig, two sequencing libraries, namely, m^6^A-Seq (IP) and RNA-Seq (Input), and the results were analyzed bioinformatically. By MeRIP-Seq, we detected a large number of m^6^A methylation peaks in the transcriptome of the muscle tissues of Queshan Black pigs, with 613 DMPs were detected. In this study, through GO, KEGG and GSEA, it is speculated that DEGs and DMGs have potential important functions in the regulation of skeletal muscle development and are involved in many important pathways, such as AMPK signaling pathway, FoxO signaling pathway, PI3K-Akt signaling pathway and Wnt signaling pathway. Among them, the AMPK signaling pathway is an important signaling pathway of energy metabolism that is heavily involved in skeletal muscle metabolic processes; controls skeletal muscle growth and development by regulating many downstream targets; is related to myocyte energy metabolism, protein synthesis, and catabolism; and plays an important role in regulating muscle mass and regeneration [39]. The MAPK signaling pathway is a class of transcription factors that promote skeletal muscle development, and genes on this pathway are continuously expressed from myogenic cell development to myotube formation. The Wnt signaling pathway is an important pathway that regulates skeletal muscle development and differentiation. It is mainly reflected in myogenic regulatory factors (MRFs) gene family myogenic regulatory factor 5(*Myf5*) and myogenic determination gene (*MyoD*) in the early stage of myogenesis [40]. The PI3K–Akt signaling pathway is involved in the regulation of important biological processes, such as muscle growth and development, metabolic regulation and homeostasis maintenance. The aberrant expression of genes related to the PI3K–Akt signaling pathway or the abnormal phosphorylation of proteins can be detected in diseases, such as antimyotrophic protein-deficient muscle and Duchenne muscular dystrophy [41]. Members of the Fox family, especially the *FOXO1* gene, are closely associated with muscle growth and development, as they play a key role in myogenic cell fusion and myofiber type conversion [42]. PPAR is a member of the nuclear receptor transcription factor family involved in growth and development, metabolism, inflammation, and many cellular processes in different organs; three PPAR isoforms, namely, PPARα, -β/δ, and -γ, are expressed in skeletal muscle in different degrees, but all have a remarkable impact on muscle homeostasis, directly or indirectly [43].

Skeletal muscle development is a complex biological process that is regulated by multiple mechanisms. The study of the regulatory role of myogenic regulators and the regulation of skeletal muscle development through epigenetic modifications has provided a preliminary understanding of the regulatory network of skeletal muscle development [44]. Based on previous studies on m^6^A methylation [35], m^6^A is hypothesized to be involved in the regulation of skeletal muscle development process. m^6^A-related modifying enzymes are able to regulate muscle development by regulating the expression of genes related to muscle cell differentiation. *METTL3* is a key regulatory factor of skeletal muscle differentiation, and participates in the process of skeletal muscle differentiation of myogenic progenitor cells by regulating the mRNA expression level of myogenic transcription factors such as *MYOD* in myoblasts [45]. *METTL14* is involved in the regulation of skeletal muscle differentiation, and *METTL14* knockdown leads to the decreased expression of *MYOD* and *MYOG* in C2C12 cells, inhibiting cell differentiation and promoting cell proliferation [46]. *FTO* positively regulates the mTOR–PGC-1α pathway by affecting mTOR activity through m^6^A demethylase activity, participating in skeletal muscle differentiation [47]. In this study, m^6^A methylation was analyzed in LD muscle tissue of Chinese native breed Queshan Black pig at both newborn and adult stages. The experimental results showed that the expressions of *METTL3*, *METTL14*, *FTO*, *ALKBH5*, *YTHDF2* and *YTHDF3* in QN were higher than those in QA, indicating that methylation may play a regulatory role in the early growth stage, thus mediating the growth and development process of muscle. Subsequently, four DEGs (*IGF1R*, *CCND2*, *MYOD1* and *FOS*) and four DMGs (*CCND2*, *PHKB*, *BIN1* and *FUT2*) were selected as candidate genes through a series of analysis, and were verified by experiments.

From the above analysis, it was found that some DEGs with methylation modification and DMGs were involved in the pathways related to muscle development, may play a regulatory role in the process of muscle growth and development. Some DEGs modified by m^6^A methylation were found to be involved in pathways related to muscle development, and they play a regulatory role in muscle growth and development. *MYOD* is a member of the MRF family; myogenic differentiation 1 (*MYOD1*) promotes muscle growth and development, enhances muscle cell metabolism, and plays an important role in lean muscle mass improvement and meat quality enhancement [48]. *FOS* is enriched in the MAPK signaling pathway; is involved in cell growth and differentiation and muscle production. It can regulate skeletal muscle cell proliferation, differentiation, and transformation [49]. Insulin-like growth factor 1 receptor (*IGF1R*) is an important component of the insulin-like growth factor (IGF) system that is widely expressed in animal muscles and is a key gene affecting growth and development [50]. *IGF1R* can regulate the *MYOD* activation, promote muscle differentiation through Akt signaling, and induce *MYOG* expression to stimulate the terminal differentiation of myogenic cells [51]. As a cyclin, Cyclin D2 (*CCND2*) is involved in a number of pathways related to muscle growth and development. It has been reported that *CCND2* can significantly enhance the myogenic differentiation of muscle progenitor cells and is an effective regulator of muscle fiber generation [52]. Phosphorylase b kinase (*PHKB*) affects the disorder of skeletal muscle glycogen metabolism and is involved in the process of glycogen decomposition, which leads to the occurrence of related myopathy [53]. Lack of Fucosyltransferase 2 (*FUT2*) increases energy expenditure and heat production in brown adipose tissue [54]. Bridging Integrator 1 (*BIN1*) is a key player in muscle development and its muscle-specific isoforms are required for skeletal muscle development and function at birth and muscle regeneration in adulthood [55].

From the above analysis, it can be seen that the genes screened in this study participate in several pathways related to muscle growth and development, and all exist in the LD muscle tissue of Queshan Black pigs. Meanwhile, the regulation level of m^6^A is negatively correlated with the transcription level in muscle, indicating that m^6^A modification not only participates in the process of muscle growth and development, but also may regulate gene expression level. These results laid a foundation for further exploration of the role of m^6^A modification in muscle growth and development.

## Conclusion

This study was the first to discover the transcriptome-wide m^6^A methylation modification pattern affecting skeletal muscle development in Queshan Black pigs. The m^6^A map revealed the distribution characteristics of m^6^A modification in the transcriptome of Queshan Black pigs. A total of 1,874 DEGs and 613 DMPs were identified, including 176 up-regulated and 437 down-regulated peaks. Through bioinformatics analysis, four DEGs (*IGF1R*, *CCND2*, *MYOD1* and *FOS*) and four DMGs (*CCND2*, *PHKB*, *BIN1* and *FUT2*), which are closely related to skeletal muscle development, were selected as candidate genes for verification. The results of this study can lay a foundation for further determining the potential effect of m^6^A RNA modification on the regulation of muscle growth of Queshan Black pig, and provide a theoretical reference for the optimization of this breed.

## Supplementary Information

**Additional file 1:** **Table S1.** The primers used for the validation.

**Additional file 2:** **Table S2.** Summary of reads quality control.

**Additional file 3:** **Table S3.** Summary of reads mapped to the Sus scrofa.

**Additional file 4:** **Table S4.** Differentially methylated peaks (DMPs) in QA vs. QN.

**Additional file 5:** **Table S5.** All GO terms for the DMGs.

**Additional file 6:** **Table S6.** All KEGG pathways for the DMGs. 

**Additional file 7:** **Table S7.** Differentially expressed genes (DEGs) in QA vs QN.

**Additional file 8:** **Table S8.** All GO terms for the DMEGs. 

**Additional file 9:** **Table S9.** All KEGG pathways for the DEGs.

**Additional file 10:** **Table S10.** Intersection of DMGs and DEGs.

**Additional file 11:** **Table S11.** All GO terms for the intersection of DMGs and DEGs.

**Additional file 12:** **Table S12.** All KEGG pathways for the intersection of DMGs and DEGs.

**Additional file 13:** **Figure S1.** Refer to the genome to compare the regional distribution.

## Data Availability

All raw data of high-throughput sequencing have been deposited to the National Genomics Data Center (NGDC, https://bigd.big.ac.cn) with the dataset accession number CRA009120.
