# Supplementary material for: Transcriptome-wide analysis of RNA m6A methylation regulation of muscle development in Queshan Black pigs
Source: BMC Genomics. 2023 May 4;24:239. doi: 10.1186/s12864-023-09346-w (PMC10161540; doi:10.1186/s12864-023-09346-w)
Supplement: Supplementary file 1 — Additional file 1: Table S1. The primers used for the validation. [file 12864_2023_9346_MOESM1_ESM.docx]

**Table S1.** The primers used for the validation.

| **Primer Name** | **Sequence(5'to3')** | **Tm/℃** | **Product Size/bp** |
| --- | --- | --- | --- |
| GAPDH-F | GCCAAAAGGGTCATCATCTC | 53.6 | 287 |
| GAPDH-R | GTAGAGGCAGGGATGATGTTC | 55.2 |  |
| IGF1R-F | CCGACGAGTGGAGAAATCTG | 58.09 | 90 |
| IGF1R-R | CTCGATCACCGTGCAGTTCT | 60.11 |  |
| CCND2-F | CACCAACACCGACGTGGATT | 60.88 | 159 |
| CCND2-R | AGGTCAATATCCCGCACGTC | 59.9 |  |
| MYOD1-F | GTTCCGACGGCATGATGGAT | 60.53 | 82 |
| MYOD1-R | TCGCTGTAATAGGTGCCGTC | 59.9 |  |
| FOS-F | CGTCAATGCGCAGGACTTCT | 61.02 | 221 |
| FOS-R | CTGGCATGGTCTTCACGACT | 60.04 |  |
| m6A-CCND2-F | CACGGTTGGAGGAAGAAGCA | 60.25 | 70 |
| m6A-CCND2-R | CCCATTGGACACGGGTGATT | 60.32 |  |
| m6A-PHKB-F | GCTTCTCCCTCATGGGTTCTA | 58.88 | 98 |
| m6A-PHKB-R | CCACTCATCTACCCCAGACTGTA | 60.63 |  |
| m6A-BIN1-F | CTGGTTGGCAAGGGACTAGG | 60.04 | 76 |
| m6A-BIN1-R | TAGGAGCCACCTTCTCGACC | 60.68 |  |
| m6A-FUT2-F | CTACCTGGCCAATTACACGC | 58.99 | 99 |
| m6A-FUT2-R | AGGTCTGCCTCGATCCCAAT | 60.69 |  |
| METTL3-F | GGAACACTGCTTGGTTGGTG | 59.61 | 83 |
| METTL3-R | GAACCTCGGCTACGATCACA | 59.55 |  |
| METTL14-F | GAGATTGCAGCTCCTCGATCA | 59.93 | 88 |
| METTL14-R | CCCACTTGCGTAAACACACTC | 59.74 |  |
| FTO-F | GATCTCAATGCCACCCACCA | 60.03 | 237 |
| FTO-R | CCACTCAAACTCGACCTCGT | 59.69 |  |
| ALKBH5-F | TGCAAGTTCCAGTTCAAGCC | 58.97 | 180 |
| ALKBH5-R | CGCATCTAACCTTGTCTTCCTGAG | 61.21 |  |
| YTHDF2-F | CAGGCAAGGCCCAATAATGC | 59.89 | 167 |
| YTHDF2-R | TCTCCGTTGCTCAGTTGTCC | 59.97 |  |
| YTHDF3-F | GCAGCCAACTGCTAGACCTAA | 60.07 | 177 |
| YTHDF3-R | TTCGCCTGTTTCCTCTGCTC | 60.32 |  |
