# Supplementary material for: Transcriptome-wide analysis of RNA m6A methylation regulation of muscle development in Queshan Black pigs
Source: BMC Genomics. 2023 May 4;24:239. doi: 10.1186/s12864-023-09346-w (PMC10161540; doi:10.1186/s12864-023-09346-w)
Supplement: Supplementary file 2 — Additional file 2: Table S2. Summary of reads quality control. [file 12864_2023_9346_MOESM2_ESM.docx]

**Table S2.** Summary of reads quality control.

| **Sample_ID** | **Raw_Reads** | **Raw_Bases** | **Valid_Reads** | **Valid_Bases** | **Valid%** | **Q20%** | **Q30%** | **GC%** |
| --- | --- | --- | --- | --- | --- | --- | --- | --- |
| QA1_IP | 49106920 | 7.37G | 46034178 | 6.32G | 85.84 | 97.54 | 93.30 | 49.88 |
| QA2_IP | 48959660 | 7.34G | 46335660 | 6.38G | 86.88 | 97.47 | 93.16 | 49.12 |
| QA3_IP | 44915212 | 6.74G | 42562604 | 5.86G | 86.96 | 97.37 | 93.02 | 49.99 |
| QA1_input | 47595794 | 7.14G | 45101400 | 6.25G | 87.59 | 97.68 | 93.42 | 50.93 |
| QA2_input | 46963436 | 7.04G | 45105214 | 6.27G | 88.99 | 97.68 | 93.39 | 50.68 |
| QA3_input | 47042758 | 7.06G | 44955654 | 6.25G | 88.51 | 97.64 | 93.34 | 50.73 |
| QN1_IP | 40096068 | 6.01G | 37908304 | 5.22G | 86.76 | 97.53 | 93.21 | 48.29 |
| QN2_IP | 43828638 | 6.57G | 42182856 | 5.80G | 88.23 | 97.42 | 92.98 | 48.48 |
| QN3_IP | 44801926 | 6.72G | 43193354 | 5.95G | 88.53 | 97.49 | 93.14 | 48.26 |
| QN1_input | 42621290 | 6.39G | 40958540 | 5.68G | 88.82 | 97.60 | 93.21 | 48.61 |
| QN2_input | 36084438 | 5.41G | 34600216 | 4.80G | 88.75 | 97.50 | 92.97 | 48.54 |
| QN3_input | 44750478 | 6.71G | 43527860 | 6.05G | 90.10 | 97.52 | 93.05 | 48.93 |
