# Supplementary material for: Transcriptome-wide analysis of RNA m6A methylation regulation of muscle development in Queshan Black pigs
Source: BMC Genomics. 2023 May 4;24:239. doi: 10.1186/s12864-023-09346-w (PMC10161540; doi:10.1186/s12864-023-09346-w)
Supplement: Supplementary file 3 — Additional file 3: Table S3. Summary of reads mapped to the Sus scrofa. [file 12864_2023_9346_MOESM3_ESM.docx]

**Table S3.** Summary of reads mapped to the *Sus scrofa*.

| **Sample** | **Valid reads** | **Mapped reads** | **Unique Mapped reads** | **Multi Mapped reads** |
| --- | --- | --- | --- | --- |
| QA1_IP | 40752050 | 37583588 (92.23%) | 27682916 (67.93%) | 9900672 (24.29%) |
| QA2_IP | 40121586 | 37036028 (92.31%) | 27408567 (68.31%) | 9627461 (24.00%) |
| QA3_IP | 36902436 | 33808459 (91.62%) | 24910862 (67.50%) | 8897597 (24.11%) |
| QN1_IP | 30651320 | 28666656 (93.53%) | 22167313 (72.32%) | 6499343 (21.20%) |
| QN2_IP | 32705866 | 30605068 (93.58%) | 23359524 (71.42%) | 7245544 (22.15%) |
| QN3_IP | 34868200 | 32638689 (93.61%) | 24480373 (70.21%) | 8158316 (23.40%) |
| QA1_input | 41576978 | 39470115 (94.93%) | 27407263 (65.92%) | 12062852 (29.01%) |
| QA2_input | 41019200 | 38894523 (94.82%) | 27552562 (67.17%) | 11341961 (27.65%) |
| QA3_input | 40331310 | 38228008 (94.78%) | 27059321 (67.09%) | 11168687 (27.69%) |
| QN1_input | 33537230 | 31708786 (94.55%) | 24070258 (71.77%) | 7638528 (22.78%) |
| QN2_input | 26798852 | 25340463 (94.56%) | 19022862 (70.98%) | 6317601 (23.57%) |
| QN3_input | 36533056 | 34639264 (94.82%) | 25278032 (69.19%) | 9361232 (25.62%) |
