# Supplementary material for: Transcriptome-wide analysis of RNA m6A methylation regulation of muscle development in Queshan Black pigs
Source: BMC Genomics. 2023 May 4;24:239. doi: 10.1186/s12864-023-09346-w (PMC10161540; doi:10.1186/s12864-023-09346-w)
Supplement: Supplementary file 13 — Additional file 13: Figure S1. Refer to the genome to compare the regional distribution. [file 12864_2023_9346_MOESM13_ESM.docx]

**Fig. S1** Refer to the genome to compare the regional distribution.
